# Glucose uptake in trophoblasts of GDM mice is regulated by the AMPK-CLUT3 signaling pathway

**DOI:** 10.1038/s41598-024-61719-7

**Published:** 2024-05-27

**Authors:** Zhenghua Xiao, Xue Liu, Xiaojin Luan, Ran Duan, Wei Peng, Chao Tong, Juan Qiao, Hongbo Qi

**Affiliations:** 1 Department of Obstetrics, Yongchuan Hospital of Chongqing Medical University, Chongqing, 402160 People’s Republic of China; 2 Chongqing Key Laboratory of Maternal and Fetal Medicine, The First Affiliated Hospital of Chongqing Medical University, Chongqing, 400016 People’s Republic of China; 3 Department of Obstetrics, The First Affiliated Hospital of Chongqing Medical University, Youyi Road, Yuzhong District, Chongqing, 400016 People’s Republic of China; 4 Women and Children’s Hospital of Chongqing Medical University, Chongqing, 401147 People’s Republic of China

**Keywords:** Gestational diabetes mellitus, GLUT3, AMPK, Glucose uptake, AICAR, Intrauterine growth, Endocrinology, Nanoscience and technology

## Abstract

GDM, as a metabolic disease during pregnancy, regulates GLUT3 translocation by AMPK, thereby affecting glucose uptake in trophoblasts. It provides a new research idea and therapeutic target for alleviating intrauterine hyperglycemia in GDM. STZ was used to construct GDM mice, inject AICAR into pregnant mice, and observe fetal and placental weight; flow cytometry was employed for the detection of glucose uptake by primary trophoblast cells; immunofluorescence was applied to detect the localization of GLUT3 and AMPK in placental tissue; Cocofal microscope was used to detect the localization of GLUT3 in trophoblast cells;qRT-PCR and Western blot experiments were carried out to detect the expression levels of GLUT3 and AMPK in placental tissue; CO-IP was utilized to detect the interaction of GLUT3 and AMPK. Compared with the normal pregnancy group, the weight of the fetus and placenta of GDM mice increased (P < 0.001), and the ability of trophoblasts to take up glucose decreased (P < 0.001). In addition, AMPK activity in trophoblasts and membrane localization of GLUT3 in GDM mice were down-regulated compared with normal pregnant mice (P < 0.05). There is an interaction between GLUT3 and AMPK. Activating AMPK in trophoblasts can up-regulate the expression of GLUT3 membrane protein in trophoblasts of mice (P < 0.05) and increase the glucose uptake of trophoblasts (P < 0.05). We speculate that inhibition of AMPK activity in GDM mice results in aberrant localization of GLUT3, which in turn attenuates glucose uptake by placental trophoblast cells. AICAR activates AMPK to increase the membrane localization of GLUT3 and improve the glucose uptake capacity of trophoblasts.

## Introduction

Gestational diabetes mellitus(GDM) is one of the most common complications during pregnancy, affecting about 8–25% of pregnancies^1^. The overall prevalence of GDM in mainland China is about 14.8%, and it has continued to increase in the past 10 years^2^.GDM refers to a condition in which women without diabetes develop diabetes during pregnancy, which usually occurs in the middle or late pregnancy and is related to insulin resistance, hyperglycemia, and maternal hyperinsulinemia^3,4^. During early gestation, insulin sensitivity increases to regulate glucose metabolism and store nutrients for later energy needs. However, maternal insulin sensitivity declines progressively in the second trimester, resulting in overt insulin resistance in the third trimester of pregnancy^5^. Hyperinsulinemia as a means of compensating for insulin resistance, through the pancreas β Cells release more insulin to cope with insulin resistance or metabolic stress^6^. Insufficient β-cell compensation for maternal insulin resistance contributes to reduced insulin secretion and impaired glucose tolerance, characteristic of GDM^7^.GDM may have an adverse effect on the health of mothers and offspring. Women with GDM are at an increased risk for experiencing pregnancy complications such as miscarriage, dystocia, preeclampsia, fetal malformations, perinatal macrosomia, and neonatal hypoglycemia^8,9^. In addition, GDM may also increase the risk of type 2 diabetes, cardiovascular disease, childhood obesity, etc^10,11^. Despite these implications, the exact pathogenesis of GDM remains unclear, resulting in a lack of targeted therapeutic interventions for its underlying cause.

Glucose, as the main energy substrate, is essential for the growth and development of the fetus and placenta. During pregnancy, the glucose transporters(GLUTs) transport glucose from the maternal circulation to the fetus by facilitated diffusion in the placenta^12,13^. The GLUTs family includes 14 subtypes, which transport glucose and other nutrients across different tissues^14^. Studies have shown that GLUT-1, GLUT-3, GLUT-4, GLUT-8, and GLUT-12 have been detected in human placental tissues, among which GLUT1, GLUT3, and GLUT4 have been reported as the main glucose transporters^15–17^. GLUT4, a glucose transporter sensitive to insulin, is mainly distributed in adipose tissues, myocardium, and skeletal muscle, and its activity is regulated by the insulin signaling pathway^18,19^. Studies have found a decrease in GLUT4 expression in the placental tissue of GDM women^20^. The glucose metabolism of trophoblasts is possibly regulated through the IRS1/GLUT4 signaling pathway in mice fed with a high-fat diet after FGF(fibroblast growth factor) is given^21^. Currently, glucose uptake and transport in the placenta are primarily mediated by GLUT1 and GLUT3^22^. GLUT1 is widely expressed in placental tissues and is responsible for basic glucose transport^23^. In contrast, GLUT3 exhibits a more restricted expression pattern, specifically in placental trophoblasts, and demonstrates a high affinity for glucose compared to other monosaccharides such as mannose, xylose, and galactose. This suggests that GLUT3 plays a unique role in facilitating glucose transport in the placenta^22,24^. Studies have shown that in the mouse model of Intrauterine Growth Restriction (IUGR), the expression level of placental GLUT3 is down-regulated, and the glucose transported through the placenta is also reduced^25^. The above proofs indicate that the changes of GLUT3 in placental tissue may play an essential role in glucose uptake and metabolism.

Adenosine 5 ‘-monophosphate-activated protein kinase(AMPK) acts as an essential energy sensor, which can be activated to promote decomposition metabolism and reduce synthetic metabolism. AMPK is a relatively complex heterotrimeric serine/threonine kinase, consisting of a catalytic (A1, A2) subunit and two regulation (B1, B2, and γ1, gamma 2 or γ3) subunits^26^. AMPK activation occurs through the phosphorylation of threonine at site 172 on the A2 subunit. AMPK controls the energy metabolism and glucose steady state of skeletal muscle, liver, adipose tissue, and pancreatic β cells. The AMPK pathway is the major target in preventing many metabolic diseases, including cancer, fatty liver diseases, and diabetes^27–29^. Evidence suggests that activation of AMPK in skeletal muscle cells can promote GLUT4 film transition, consequently increasing glucose intake^30^. However, a few studies focused on the relationship between AMPK and GLUT3 have found that AMPK in the activation of colon cancer cells can promote GLUT3 expression, and the level of GLUT3 is significantly up-regulated in the use of the AMPK agonist 5-aminoimidazole-4-carboxamide ribonucleotide (AICAR)^31^. However, whether the AMPK-GLUT3 regulatory axis exists in the mouse placenta remains unclear. Therefore, in this study, an animal model of GDM was constructed and AICAR was used to stimulate pregnant mice to study the possible role of AMPK-GLUT3 in mouse placental tissue.

## Materials and methods

### Chemicals and assay kits

BCA PROTEIN ASSAY KIT,MEMBRANE AND CYTOSOL PROTEIN EXTRACTION KIT,TRYPSIN–EDTA SOLUTION,BEYOCOLOR PRESTAINED COLOR PROTEIN MARKER,SDS-PAGE SAMPLE LOADING BUFFER,BEYOECL MOON were purchased from BEYOTIME.PHOSPHO-AMPKa (Thr172) RABBIT mAb and AMPKa ANTIBODY were acquired from CST.STREPTOZOCIN,PVDF MEMBRANCRS,PROTIEN G–AGAROSE, FOETAL BOVINE SERUM and COLLAGENASE were obtained from SIGMA.PERCOOL, SODIUM CITRATE BUFFER and PROTEINASE K were purchased from SOLARBO.GLUT3 ANTIBODY was procured from SCB.2-NBDG was bought from THERMOFISHER.AICAR was acquired from SELLECK.CN.HRP-CONJUGATED AFFNIPURE GOAT ANTI-MOUSE IgG(H + L),HRP-CONJUGATED AFFNIPURE GOAT ANTI-RABBIT IgG(H + L),FITC-CONJ UGATED AFFNIPURE GOAT ANTI-RABBIT IgG(H + L),R-PE-CONJUGATED GOAT ANTI-RABBIT IgG(H + L),CYTOKERATIN 7-SPECIFIC POLYCLONAL ANTIBODY, AMPK ALPHA MONOCLONAL ANTIBODY and ALPHA TUBULIN POLYCLONAL ANTIBODY were purchased from PROTEINTECH.ACRYL/BIS 30% SOLUTION, 1MTRIS- HClSOLUTION(pH6.8),4XTRIS-HCL/SDS(pH8.8),TEMED,TRIS–GLYCINE-SDSRUNNING BUFFER,EZ-BUFFERS C 10X WESTERN TANSFER BUFFER and EZ-BUFFERS H 10X TBST BUFFER were acquired from SANGON BIOTECH. PRIMSCRIP RT MASTER MIX and TB GREEN PREMIX EX TAQ were obtained from TAKARA.

### Animal and experimental design

Unfertilized female C57BL/6 J mice, aged 6–8 weeks, were purchased from Chongqing Tengxin Biological Co.Ltd, and then fed in the SPF animal laboratory of Chongqing Medical University. All procedures were conducted following ARRIVE guidelines.This study was approved by the Chongqing Medical University Ethics Committee. Mice were raised in the animal laboratory of Chongqing Medical University on a regular 12 h night and day cycle and fed with a standard murine diet and water ad libitum. After being fed for around 9–10 weeks, the female mice were mated with the male mice at a ratio of 1:1. Female mice were examined for the vaginal plug at 8:00 AM the following morning. The day on which a vaginal plug was detected was considered the gestation day 0.5 (GD0.5). The pregnant mice were divided into 4 groups: normal pregnancy group, GDM group, Normal + AICAR group, and GDM + AICAR group, with 3–5 mice in each group. The GDM and GDM + AICAR groups were fasted for 8 h prior to administration, and were then injected with 150 mg/kg of STZ diluted in sodium citrate at GD12.5^32,33^. while the Normal + AICAR group and GDM + AICAR group were injected with 250 mg/kg AICAR at GD17.5^34^. For the oral glucose tolerance test(OGTT), each group was given 2 mg/kg glucose at GD14.5 and GD18.5. Then, blood samples were collected from the tail vein at 0, 30, 60, 90, and 120 min to measure blood glucose levels.

### Tissue collection and preparation

All pregnant mice were euthanized at GD18.5, and then blood, offspring, and placenta were collected. Phosphate-buffered saline (PBS) was pre-cooled to wash the blood of the placenta tissue until the rinse was colorless. Part of the placenta and plasma were placed in a cryotube and stored in the refrigerator at – 80 ℃ for qRT-PCR, Western Blot experiments and Enzyme-Linked Immunosorbent Assay. The tissue sample was embedded in paraffin and stored at room temperature before immunofluorescence experiments. Primary trophoblast cells were extracted from the remaining placental for 2-NBDG uptake experiments.

### Enzyme-Linked Immunosorbent Assay(ELISA)

The ELISA assay was performed to examine the contents of insulin in the placental peripheral plasma in mice. Firstly, the standard and plasma samples at 10 ul were added to the plates. Then,100 ul labeling mouse Insulin antibodies were applied to each well, respectively.the plates were incubated the plate at 37 ℃ for 2 h. The plates were washed five times with washing buffer. The plates were incubated for another 1 h with the addition of TMB substance solution and were kept away from light. Without rinse, a stop solution was applied to all the wells. Within 10 min after the color changed, the OD values were determined with a microplate reader at a wavelength of 450 nm.The HOMA-IR was then calculated as:[fasting glucose (mmol/L)xfasting insulin (mU/L)]/22.5.

### 2-NBDG uptake assay

First, mouse placental tissue was added to 1 ml of collagenase IV at a concentration of 0.01 g/ml, quickly chopped, and then placed in a 37 °C incubator for full digestion of 30 min. Then, 5 ml of complete medium was added to stop digestion, the cell suspension was sieved with a 70 um cell strainer centrifuged at 1500 rpm for 5 min, and the supernatant was discarded and resuspended in 5 ml of stain buffer for later use. Next, percoll mixtures with concentrations of 60%, 40%, 20%, and 5% were prepared and added to a 50 ml centrifuge tube in turn. The above cell suspension was slowly added to the uppermost layer. The cell suspension at 20–40% density layer was collected after centrifugation at 1500 rpm for 5 min. Next, the mixture was resuspended in 5 ml of complete medium, and the cell pellets were collected after differential adherence for 30 min and resuspended and seeded in 24-well plates for 24 h. The next day, the 24-well plate was washed twice with pre-cooled PBS and then incubated with 100um of 2-NBDG for 30 min. Finally, cells were collected, and the intracellular fluorescence intensity was immediately measured using flow cytometry.

### RNA extraction and quantitative RT-PCR

The placenta was freshly dissected, snap-frozen, and stored at− 80 °C. Total RNA from the placenta was isolated with a TRIzol reagent. Chloroform was used for phase separation, ice-cold isopropanol was utilized for RNA precipitation, and 75% ethanol was applied to wash RNA precipitation. After centrifugation at 16,000 g for 10 min at 4 °C, pellets were resuspended in 20 μl diethyl pyrocarbonate-treated water.RNA concentration was quantified using the Nanodrop. A 1 mg RNA was reversed transcribed to cDNA using the cDNA reverse transcription kit. Each PCR reaction (20 μl final volume) was run at 37 °C for 15 min, 85 °C for 5 s, and then kept at 4 °C.cDNA were used for quantitative real-time PCR (RT-qPCR) mixed with TBGR PremixEx Taqr and primers in a total volume of 10 μl. Amplification was performed on the BioRad CFX96 real-time PCR system. The gene expression levels were normalized using the housekeeping gene GAPDH. The following is the primer sequence of each gene:

- GLUT3:Sense5′-GAGATGAAGGATGAGAGTGTTCGGATG-3’,
  - Anti-sense5’-GCTGGAGGACAATGGAGATGAGAAG-3’;
- AMPK:Sense5′-GCCTTGAAAGAAGTGTGTGAGAAGTTC-3’, Anti-sense5’-GTGGGTCCTGGTGGTTTCTGTTG-3’;
- GAPDH:Sense5′-ATGTGTCCGTCGTGGATCTGAC-3’,
- Anti-sense 5′-AGACAACCTGGTCCTCAGTGTAG-3’.

Finally, the 2-ΔΔct method was used to calculate the relative expression of sample genes.

### Protein extraction and western blot

30 mg of placental tissue was taken from each sample, RIPA lysate was used for total protein extraction and membrane protein. The extraction of cytoplasmic protein was conducted according to the kit instructions. The BCA kit was used to determine the protein concentration was determined using the BCA kit. The protein was stored in a refrigerator at − 80 °C. First, SDS-PAGE was used to separate the proteins, which were then transferred to PVDF membranes. Then, the membrane was blocked in 5% no-fat milk and hybridized overnight at 4 ℃ with primary antibodies, including AMPK, P-AMPK, GLUT3, and Tubulin. The next day, the membrane was washed 5 times with TBST and incubated with the corresponding secondary antibody for 1 h. Finally, ECL was used for chemiluminescence detection, and grayscale quantitative analysis was performed by Gel-Proanlyzer software.

### Immunofluorescence and confocal microscopy

Paraffin blocks were sliced, dewaxed, and hydrated**.** PBS-rinsed sections were incubated with proteinase K for 15 min for antigen retrieval. Sections were washed 3 times with PBS for 10 min each and then added 0.3% Triton-100 for 30 min. Sections were hybridized with primary antibody overnight at 4 °C. The primary antibodies include AMPK, GLUT3, CK7, and Na + -K + -ATPase. Next, the sections were washed 3 times with PBS again, and incubated with the corresponding secondary antibodies for 1 h. Finally, Hoechst 33,442 was added and incubated with the sections for 10 min, and Fluorescence microscopy or confocal microscopy was performed to observe the fluorescent signal of the tissue on the slice.

### Co-Immunoprecipitation

For each sample, 30 mg of mouse placental tissue was placed in 1 ml of RIPA lysis buffer and lysed on ice for 30 min. Then, 200 ul supernatant of the lysate was collected after centrifuging at 16000 g at 4 °C for 30 min, and stored at − 80 °C. 30 ul protein A agarose beads were washed 3 times in 500 ul PBS on a shaker. Next, the washed Protein A agarose beads and lysate were mixed with 2ul of AMPK or GLUT3 primary antibody and rotated at 4 °C overnight. The next day, the mixture was centrifuged at 3000 rpm at 4 °C for 3 min, the supernatant was discarded, and the pellet was washed 3 times with 500 ul of PBS. Finally, the pellet was resuspended in 30 ul of RIPA lysis buffer, and 10 ul of loading buffer was added and boiled at 100 °C for 10 min. The expression levels of AMPK or GLUT3 in each protein sample were detected by western blot.

### Statistics

All statistical analysis data were analyzed using either a two-tailed independent T-Test or two-way ANOVA, as appropriate. Levene’s test was used to test for equality of variance between groups. Welch’s T-test was used for significance whenever the variance was unequal. At least three independent replicates for each experiment. Data are presented as the mean ± SEM for variables with a normal distribution and as the median and interquartile range for variables with a non-normal distribution. *P* < 0.05 is considered statistically significant. *P < 0.05; **P < 0.01; ***P < 0.001.

### Ethical approval

All procedures were conducted in accordance with ARRIVE guidelines. This study was approved by the Chongqing Medical University Ethics Committee.

## Results

### Decreased glucose uptake capacity of trophoblast cells in GDM mice

**Figure 1:**
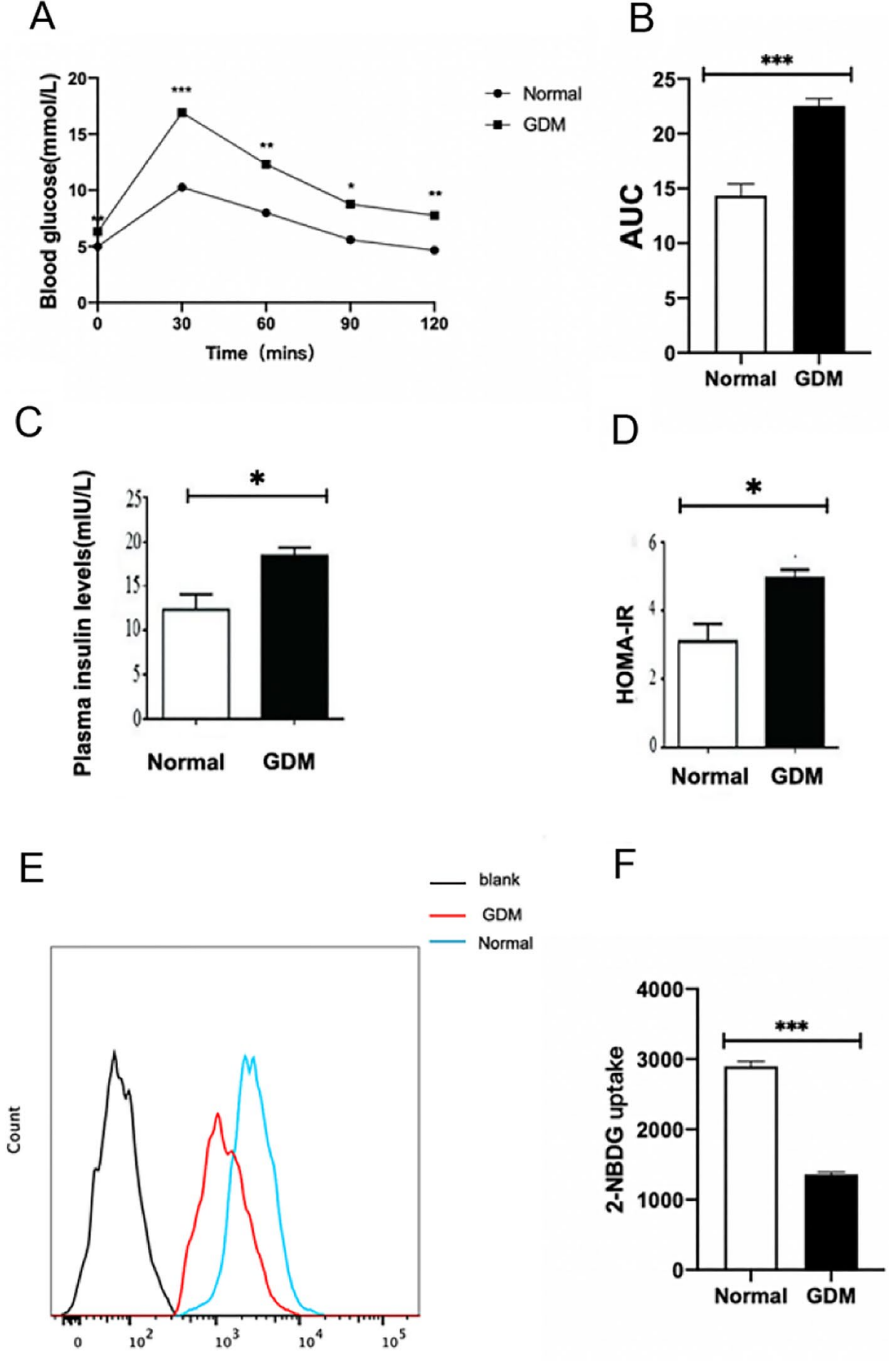
Decreased glucose uptake capacity of trophoblast cells in GDM mice. (**A**) OGTT curves following oral gavage dose of 2 g/kg D-glucose at GD14.5. (**B**) AUCs of OGTT curves at GD14.5. (**C**)The plasma insulin levels. (**D**)The HOMA-IR.(**E** **F**) Fluorescence intensity and quantitative analysis of 2-NBDG in trophoblast cells by flow cytometry at GD18.5.*P < 0.05, **P < 0.01 and ***P < 0.001 vs. normal group.

Pregnant C57 mice were injected intraperitoneally with 150 mg/kg STZ or an equal volume of PBS at GD12.5. OGTT test was performed on two groups of mice at GD14.5. As shown in Fig. 1A, the blood glucose of the mice in the GDM group was higher than that in the normal group at 0, 30, 60, 90, and 120 min after the mice were gavaged with glucose. The area under the blood glucose curve in the GDM group was also higher than in the control group (P < 0.001; Fig. 1B). The insulin levels and HOMA-IR also higer in the GDM group(P < 0.05; Fig. 1C,D).In addition, the results of the 2-NBDG experiment showed that the glucose uptake capacity of the trophoblasts in the GDM group was significantly decreased (P < 0.001; Fig. 1E,F).

### Offspring and placental weights of GDM mice were increased

**Figure 2:**
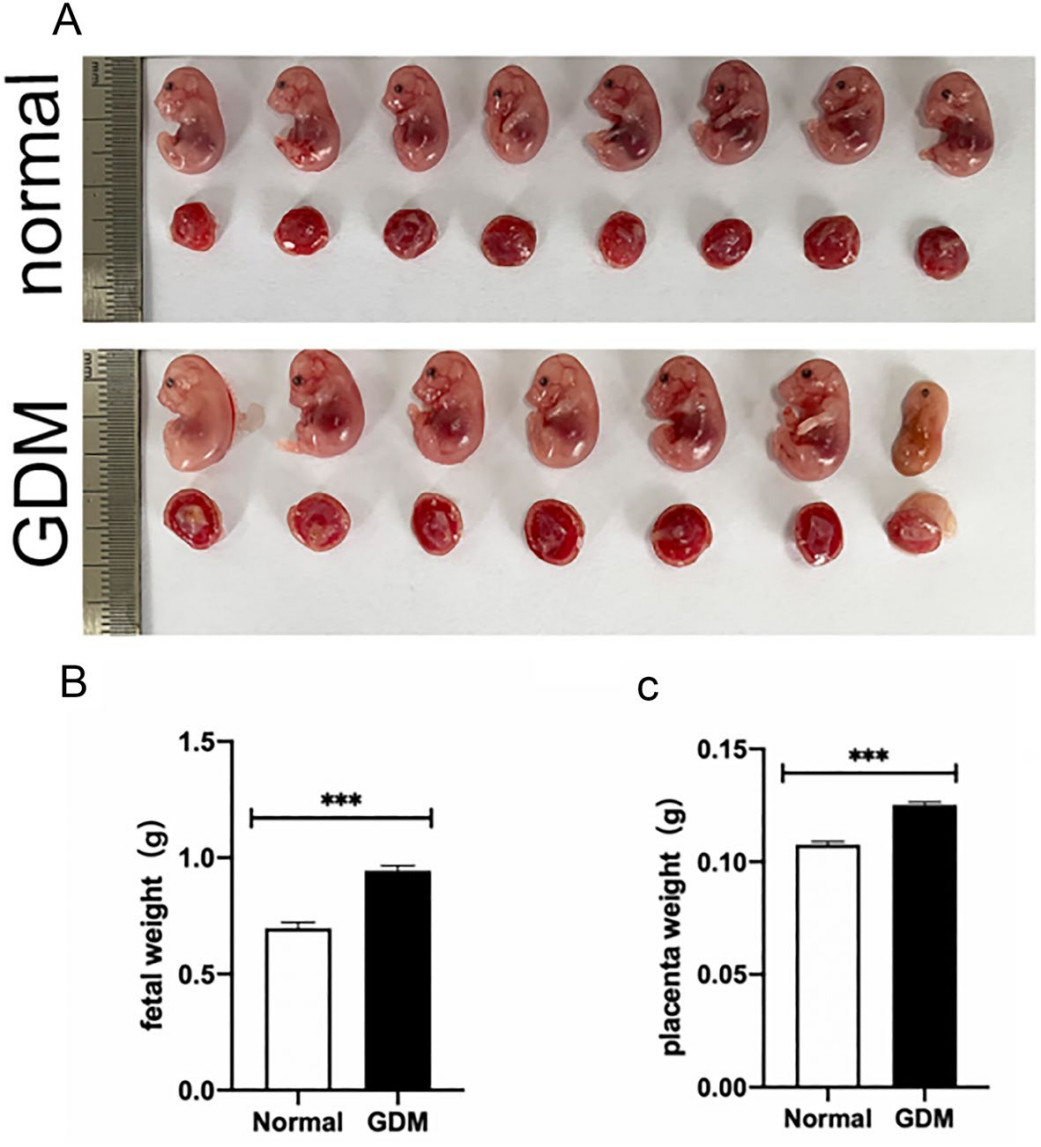
Offspring and placental weights of GDM mice were increased. (**A**) The offspring and placenta of normal and GDM mice. (**B**) Weight of offspring of mice in normal and GDM groups. (**C**) Placental weight of mice in normal and GDM groups. ***P < 0.001 vs. normal group.

Euthanized mice at GD18.5 were tested, and the offspring and placental weights of normal pregnancy and GDM mice were tested (Fig. 2A). It was found that the weight of the offspring (P < 0.001)and placenta (P < 0.001)of GDM mice were significantly higher than that of the normal pregnancy group(Fig. 2B, C).

### Down-regulation of GLUT3 expression on trophoblast cell membranes in GDM mice

**Figure 3:**
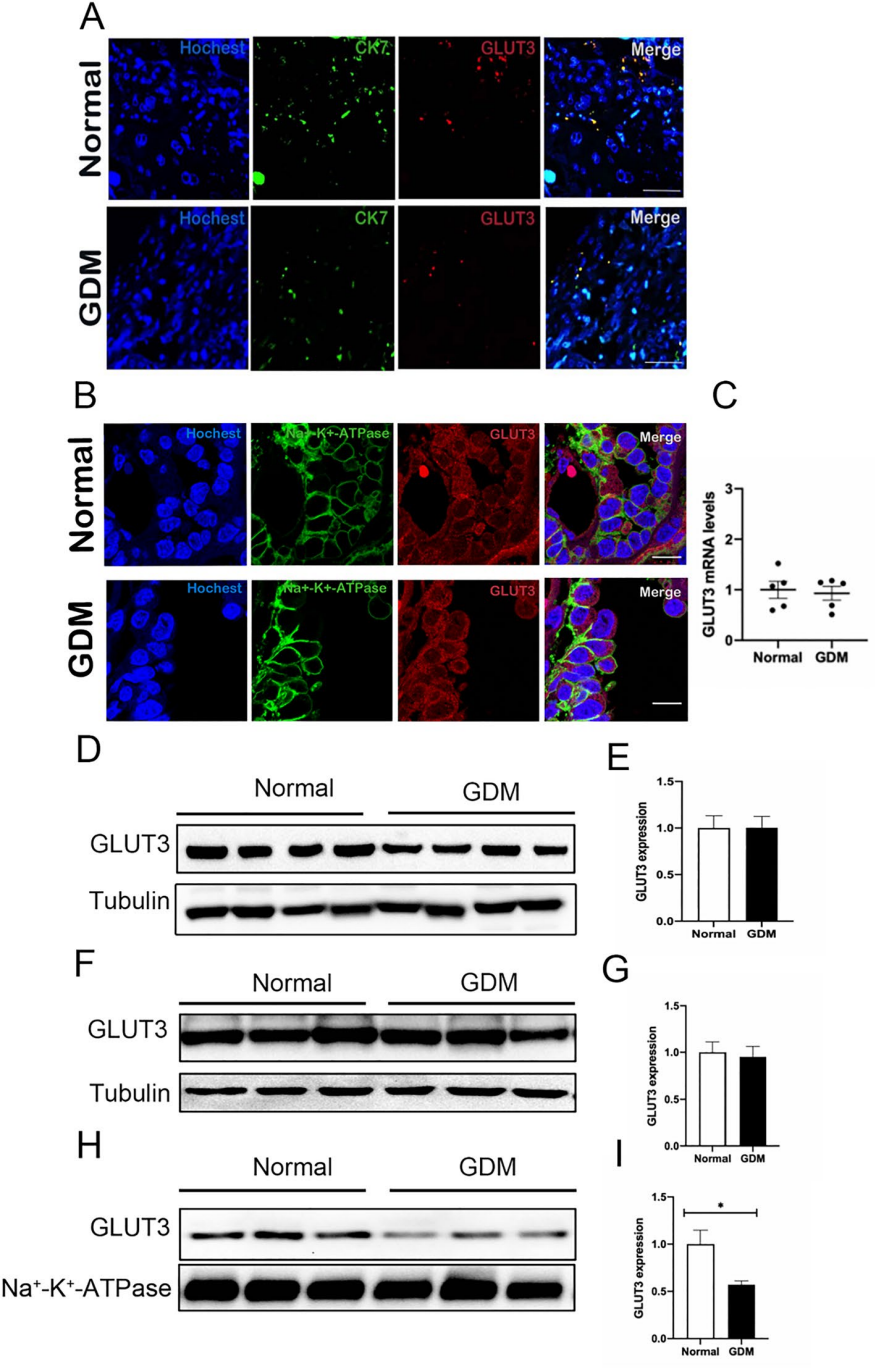
Down-regulation of GLUT3 expression on trophoblast cell membranes in GDM mice. (**A**) Representative photomicrographs of CK7 and GLUT3 expression in immunofluorescent stained normal and GDM mouse placental tissues. (**B**) Representative micrographs of GLUT3 localization and expression in confocal normal and GDM mouse placental tissues. (**C**) qRT-PCR analysis of GLUT3. (**D**, **E**) Western blot analysis of total GLUT3 protein expression. (**F**, **G**) Western blot analysis of cytoplasmic GLUT3 protein expression. (**H**, **I**)Western blot analysis of GLUT3 protein expression on cell membrane. *P < 0.05,vs normal group.

We found that CK7 and GLUT3 co-localized in the placental tissue of the normal and GDM groups, and the proportion of GLUT3-positive cells in the GDM group decreased by immunofluorescence (Fig. 3A). Confocal microscopy imaging showed that GLUT3 is expressed in the cell membrane and cytoplasm of both the normal and GDM groups(Fig. 3B). Meanwhile, the qRT-PCR experiment showed no significant difference in the transcription level of GLUT3 in the placenta tissue of the two groups (P > 0.05, Fig. 3C). Previous studies have shown that GLUT3 exhibits rapid and significant membrane translocation during a marked increase in neuronal glucose uptake. Interestingly, western blot results suggested no difference in the expression levels of total GLUT3 protein and cytoplasmic GLUT3 protein in the placental tissue of the normal and the GDM group (Fig. 3D,E,F,G). However, the expression level of GLUT3 protein in the placental tissue cell membranes of the GDM group was significantly decreased (P < 0.05,Fig. 3H,I), which suggested that the level of GLUT3 on the trophoblast cell membranes of GDM mice was down-regulated.

### p-AMPK was significantly down-regulated in the trophoblast cells of GDM mice

**Figure 4:**
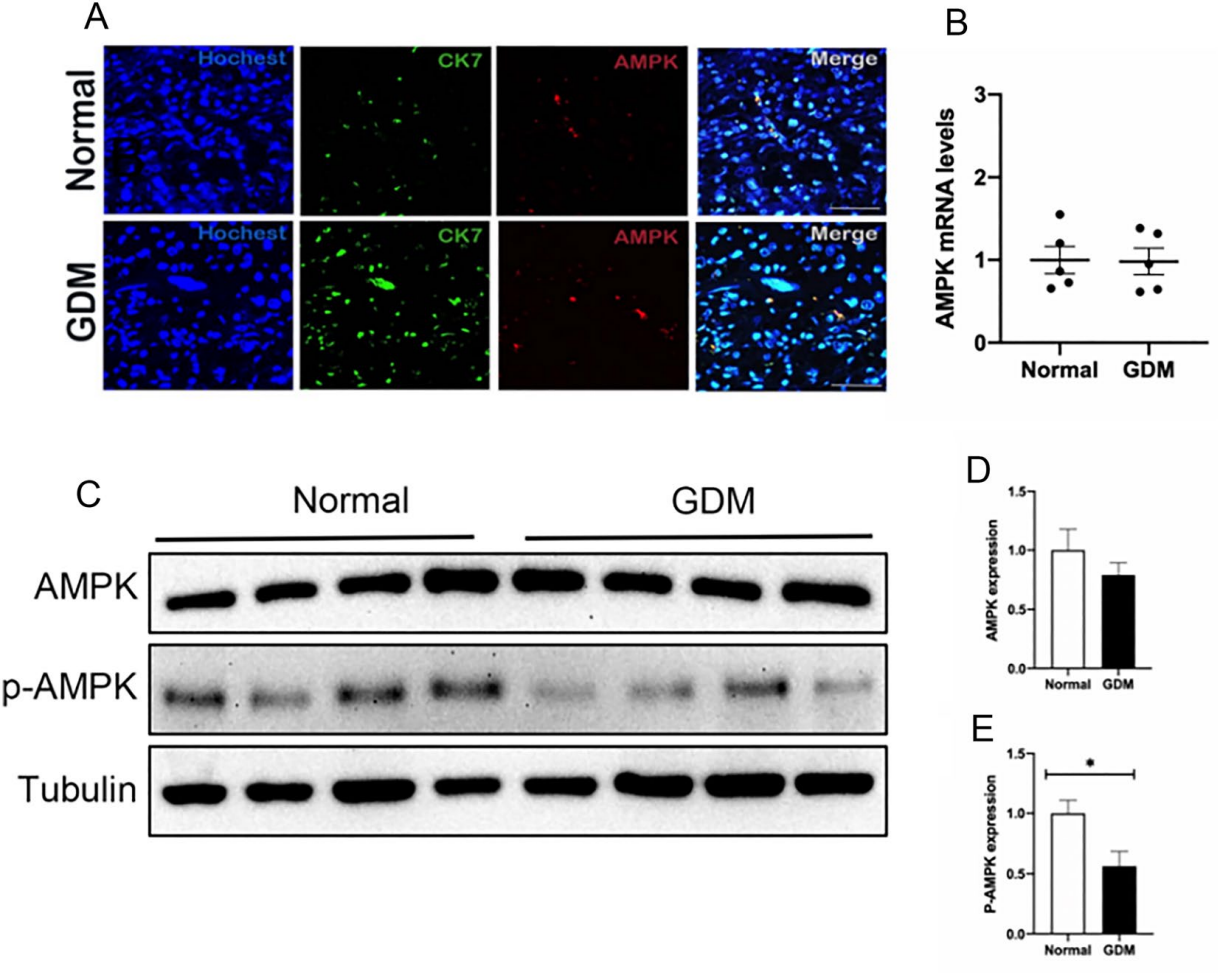
p-AMPK was significantly down-regulated in the trophoblast cells of GDM mice. (**A**) Representative photomicrographs of CK7 and AMPK expression in immunofluorescently stained normal and GDM mouse placental tissues. (**B**) qRT-PCR analysis of AMPK. (**C,D,E**) Western blot analysis of cytoplasmic AMPK and p-AMPK protein expression.*P < 0.05, vs normal group.

As a key competent molecule, activated AMPK plays an important role in glucose uptake. Immunofluorescence showed that CK7 and AMPK also co-localized in the mouse placenta (Fig. 4A), and the qRT-PCR results showed no significant difference in the transcription level of AMPK in the placenta between the two groups of mice (Fig. 4B). Phosphorylation of AMPK significantly enhances AMPK activity. Therefore, we detected the expression level of p-AMPK in placental tissue by western blot experiment. The results showed no difference in the expression level of AMPK protein in the placenta in the two groups (Fig. 4C,D), while the expression level of p-AMPK protein in the placental tissue of mice in the GDM group was significantly decreased(Fig. 4E). These results support the significant downregulation of AMPK phosphorylation in trophoblast cells of GDM mice.

### Interaction between AMPK and GLUT3 in placental tissue

**Figure 5:**
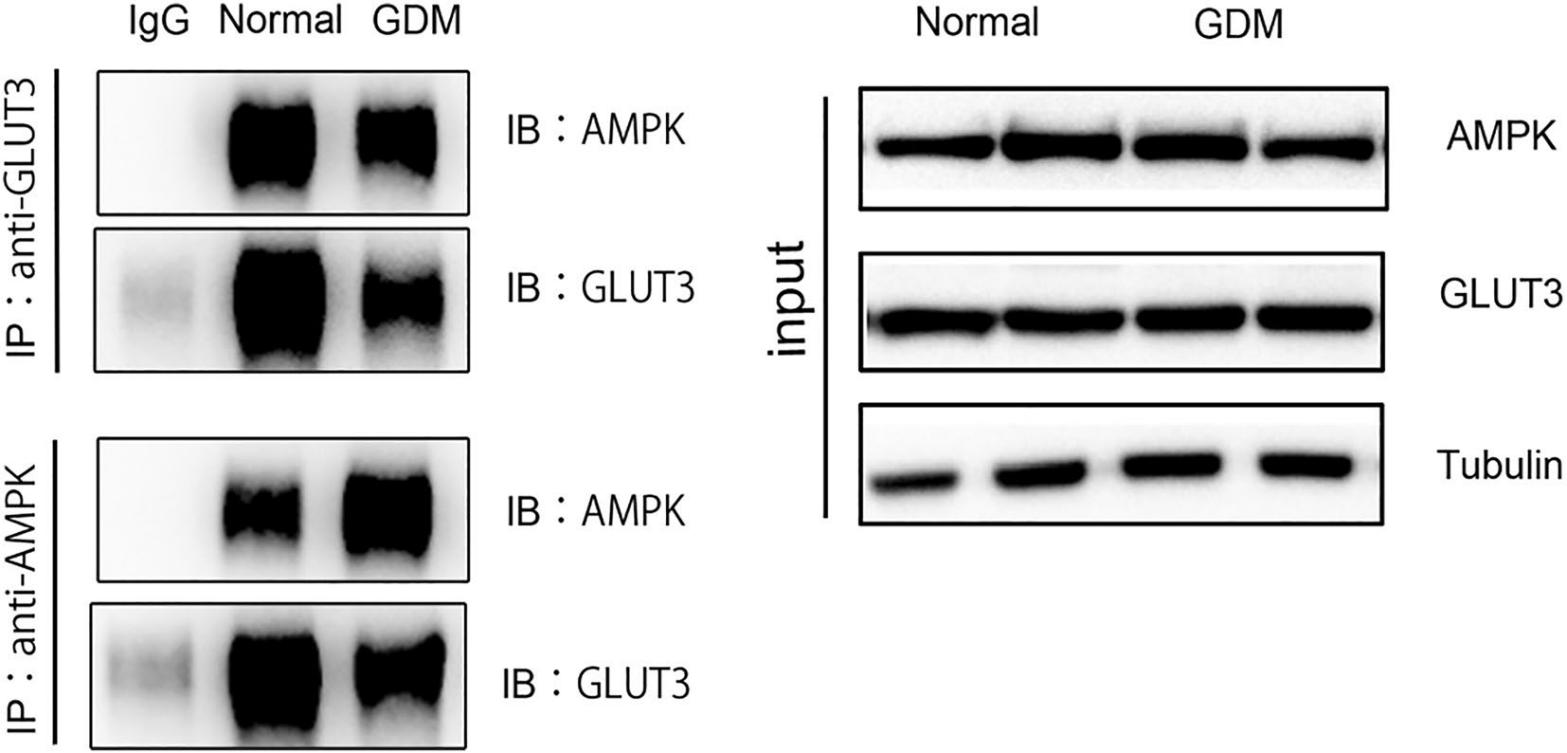
Interaction between AMPK and GLUT3 in placental tissue Interaction between AMPK and GLUT3 detected by co-immunoprecipitation.

The above evidence indicates that GLUT3, AMPK, and CK7 co-localize in mouse placenta tissue. Furthermore, the plasma membrane localization of GLUT3 was significantly down-regulated in the placental tissue of GDM mice, and the phosphorylation level of AMPK was also decreased. Previous studies have also found that activation of AMPK in neurons can promote the membrane translocation of GLUT3. Hence, our study substantiated the interaction between AMPK and GLUT3 in mouse tissue (Fig. 5).

### AICAR reduces the blood sugar level of mice and promotes the glucose uptake capacity of trophoblast cells

**Figure 6:**
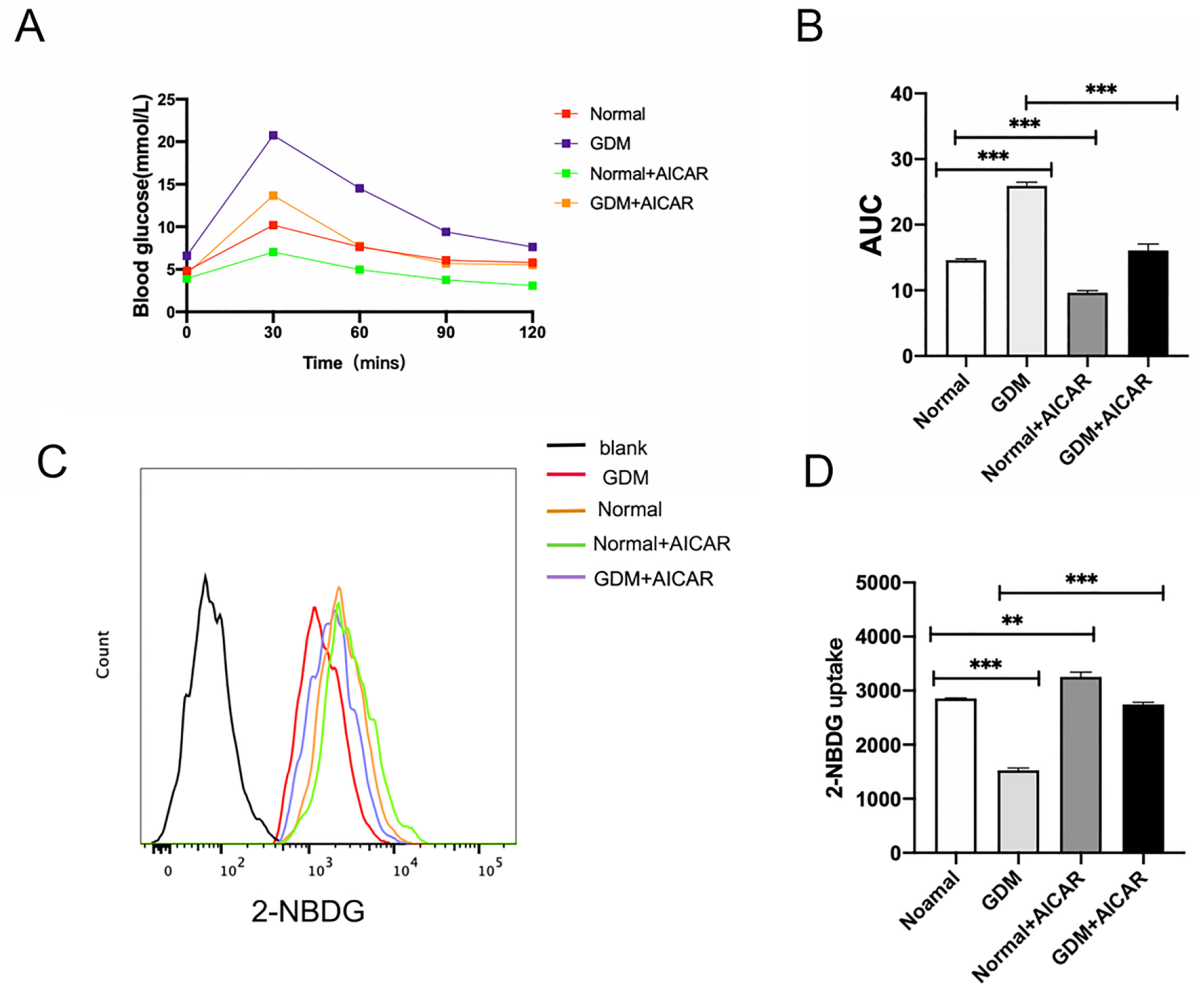
AICAR reduces the blood sugar level of mice and promotes the glucose uptake capacity of trophoblast cells. (**A**) OGTT curves following oral gavage dose of 2 g/kg D-glucose at GD18.5. (**B**) AUCs of OGTT curves at GD18.5. (**C**,**D**) Fluorescence intensity and quantitative analysis of 2-NBDG in trophoblast cells by flow cytometry at GD18.5.*P < 0.05, **P < 0.01 and ***P < 0.001 GDM group vs. normal group.GDM group vs GDM + AICAR group.Normal group vs Normal + AICAR group.

AICAR, a well-known AMPK agonist, has been shown to reduce blood sugar in mice by activating AMPK. Pregnant C57 mice were administered intraperitoneal injections with 250 mg/kg AICAR or an equivalent volume of PBS at GD17.5 to investigate this further. Subsequently, an OGTT test was performed on four groups of mice at GD18.5. The results in Fig. 1A indicated that the blood glucose levels of the mice in the AICAR group were consistently lower than those of the corresponding non-AICAR group at 0, 30, 60, 90, and 120 min following glucose administration. Furthermore, the area under the curve for blood glucose was significantly reduced after AICAR injection (P < 0.001; Fig. 6A, B). Additionally, the results of the 2-NBDG experiment showed that the glucose uptake capacity of the trophoblast cells also increased after AICAR injection(P < 0.01, Fig. 6C, D).

### AICAR up-regulated the expression levels of p-AMPK and membrane GLUT3 in mouse trophoblast cells

**Figure 7:**
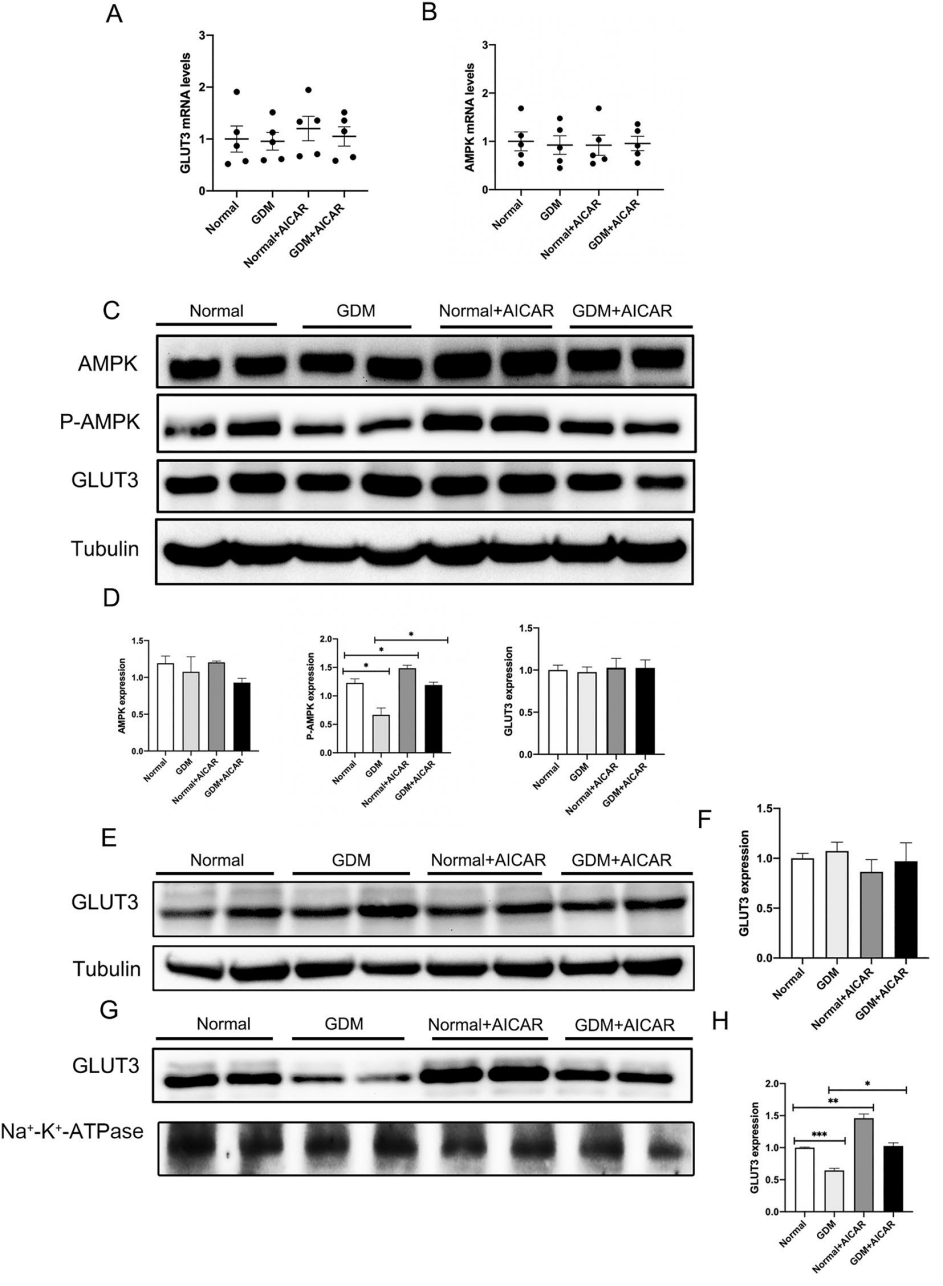
AICAR up-regulated the expression levels of p-AMPK and membrane GLUT3 in mouse trophoblast cells. (**A**, **B**)qRT-PCR analysis of GLUT3 and AMPK. (**C**,**D**)Western blot analysis of total GLUT3、AMPK、P-AMPK protein expression.(E,F)Wstern blot analysis of cytoplasmic GLUT3 protein expression. (**G**,**H**)Western blot analysis of GLUT3 protein expression on cell membrane.*P < 0.05, **P < 0.01 and ***P < 0.001 GDM group vs.normal group.GDM group vs GDM + AICAR group.Normal group vs Normal + AICAR group.

Similarly, the qRT-PCR findings indicated no substantial difference in the transcription levels of GLUT3 and AMPK in the placental tissue of the four groups of mice (Fig. 7A,B). The western Blot experiments confirmed that AICAR facilitated the phosphorylation of AMPK and the expression of GLUT3 in the mouse trophoblast cell membrane (Fig. 7C,D,G,H).In conclusion, AICAR up-regulated the expression of GLUT3 on the trophoblast cell membrane of GDM mice by activating AMPK.

## Discussion

In this study, we used STZ to construct a GDM mice model and found that compared with normal pregnant mice, the expression level of GLUT3 on the trophoblast membrane and the activity of AMPK in the trophoblast of GDM mice were significantly down-regulated.In vivo experiments have confirmed that AICAR can activate AMPK, thereby improving the abnormal localization of mice trophoblasts GLUT3 and increasing the glucose uptake of mice trophoblasts. In general, we speculate that AICAR increases the membrane localization of GLUT3 by upregulating the activity of AMPK in the trophoblasts of GDM mice, thus enhancing the glucose uptake ability of mouse trophoblast.

The transfer of glucose at the interface between the mother and fetus occurs following a glucose concentration gradient, with high concentrations in maternal blood and low concentrations in the fetus. The intrauterine hyperglycemia environment in GDM expands the glucose concentration gradient between the mother and the fetus, leading to excessive transfer of glucose from the placenta to the fetus. In turn, it affects the secretion of fetal insulin, the growth of fat cells, and the synthesis of triglycerides, ultimately leading to fetal overgrowth^35,36^. Studies have also found that GDM is related to fetal growth retardation and poor growth. An epidemiological survey in the United States shows that about 7% of the offspring of GDM females are small for gestational age(SGA)^37^.In a retrospective case–control study of 1981 SGA babies conducted in China, 383 SGA babies (19.3%) were born to mothers with GDM^38^. This study revealed a significant reduction in the glucose uptake capacity of placental trophoblasts in GDM mice compared to those in the normal pregnancy group. Additionally, notable increases was found in both fetal and placental weight in GDM mice. Furthermore, dysplasias were observed in the offspring of GDM mice. These findings suggest an imbalanced glucose metabolism in the placenta of GDM mice, thereby elevating the likelihood of adverse pregnancy outcomes.

GLUT1 and GLUT3, the major placental glucose transport proteins in humans, are widely expressed in early pregnancy and at term.GLUT1 plays a key role in. glycolysis for energy production, and its expression increases as pregnancy progresses^39^. It has been well established that the increased level of GLUT1 expression in the placenta of pregnant women with GDM is due to the increased availability of circulating glucose in the mother and the corresponding increase in glucose consumption in the placenta^40^.In contrast to studies on GLUT1, only a few studies have investigated GLUT3 changes in response to exposure to a hyperglycemic environment during pregnancy.Reported changes in GLUT3 in the placenta of women with GDM are also contradictory.Aldahmash Waleed think GLUT3 levels are increased in pregnant women with GDM.Other studies have found that GLUT3 levels are reduced in the placentas of insulin-treated women with GDM^41^.GLUT3 in mice was first found in brain tissue and showed a high expression pattern^24^. Studies have found that in the hippocampus of 3XTg-AD mice, the decrease in phosphorylation of AKT in the insulin signaling pathway leads to a decrease in GLUT3 translocation. This reduction in GLUT3 on the cell membrane may impair neurons’ ability to take up glucose^42^. Existing evidence shows that GLUT3 also plays an important role in the embryonic development of mice and the transfer of glucose from the mother to the fetus. The mouse GLUT3 mutation leads to a decrease in glucose transported through the placenta, resulting in loss of early pregnancy and fetal growth restriction in late pregnancy^43^. It can be seen that GLUT3 is an important mediator of glucose transport in the placenta^22^.In this study, we observed that the expression level of GLUT3 on the cell membrane in the placenta tissue of GDM mice was significantly lower than that of the normal pregnancy group. In contrast, the total expression level of GLUT3 in the two groups of mice did not change significantly. It shows that GLUT3 has abnormal localization in the placenta tissue of GDM mice. The intrauterine hyperglycemia environment may cause this abnormal localization.

AMPK regulates the activity of proteins in many important metabolic signaling pathways through the phosphorylation pathway and is considered a key protein related to type 2 diabetes^44^. It is worth noting that compared with the normal population, the activity of AMPK in the adipose tissue of non-pregnant obese or diabetic individuals was significantly down-regulated^45^. Although the exact mechanism of GDM is unclear, GDM is the precursor state of type 2 diabetes. GDM women have a 7.34 times higher risk of developing type 2 diabetes after delivery than normal pregnant women^46^. More and more research focuses on the mechanism of AMPK in GDM. Studies have pointed out that activating AMPK can improve inflammation and insulin resistance of skeletal muscle and adipose tissue in women with GDM^47^_._ Recent studies have shown that the presence of activated AMPK in the placental tissues of humans and mice is beneficial to the differentiation of the placenta and the growth of the fetus, the expression level of the AMPK gene in the placental tissues of obese women with GDM decreases^48,49^. Therefore, we speculate that the imbalance of glucose homeostasis in the placenta tissue of GDM mice is related to AMPK. To test this hypothesis, we constructed a GDM mice model. Although the mRNA levels of AMPK in the normal pregnancy group and GDM mice did not change significantly, Western blot experiments showed that compared with the normal pregnancy group, the phosphorylation level of AMPK in the placenta tissue of GDM mice was significantly down-regulated. Immunofluorescence showed that CK7 and AMPK also co-localized in mouse placenta. And through CO-IP detection, there is an interaction between AMPK and GLUT3 in trophoblasts. Therefore, we believe that AMPK in trophoblasts may directly interact with GLUT3 to promote the transfer of GLUT3 in the cytoplasm to the cell membrane, thereby mediating the transport of glucose, and the ingested glucose is metabolized by glycolysis for energy. The AMPK activity is inhibited in the high glucose environment caused by GDM mice, resulting in abnormal localization of GLUT3, which in turn weakens the level of glucose metabolism in the placenta itself, increases the net glucose transport from the maternal circulation to the fetal circulation, eventually affect the growth of the fetal placenta.

To further verify the above findings, in this study, in vivo experiments were conducted to investigate whether AMPK activation can regulate the translocation expression of GLUT3 and glucose transport in mice placental tissues. AICAR is a commonly used AMPK agonist, it can be absorbed and metabolized by tissues in the body into ZMP, an analog of AMP, thereby activating AMPK and promoting glucose uptake by tissues^50^. Studies have found that AICAR stimulation in rats can increase AMPK activity, thereby improving insulin sensitivity and glucose transport in rat muscles^51^.In our study, The blood glucose levels of pregnant mice at the OGTT 5 time points and the area under the curve injected with AICAR were significantly lower than those of the non-injected group. The glucose uptake capacity of placental trophoblasts also significantly increases after AICAR injection. It is suggested that AICAR can improve the impaired glucose uptake of placental trophoblasts and promote the glucose metabolism of the placenta itself. We also found that AICAR significantly increased the phosphorylation level of AMPK, and the expression level of GLUT3 on the cell membrane was also significantly increased. Therefore, we concluded that AICAR can activate AMPK and promote the translocation of GLUT3 from the cytoplasm to the cell membrane in trophoblasts, thereby regulating placental glucose homeostasis.

In conclusion, our study demonstrates that administering AICAR to STZ-induced GDM mice can stimulate trophoblast AMPK activity, thereby correcting aberrant GLUT3 localization and enhancing trophoblast glucose uptake capacity. We also give an in-depth discussion of the regulatory mechanism about glucose transport at the maternal–fetal interface, which inhibits excessive glucose transport to the fetus, reducing the adverse effects of the GDM intrauterine hyperglycemia environment on the fetus, and providing new intervention targets for improving the birth outcome of GDM. Further researches is needed to illustrate the role of AMPK-GLUT3 signal axis in regulating placental glucose homeostasis.

### Supplementary Information

Supplementary Figures.

## Data Availability

The data used to conduct this study are available from the corresponding author(Juan Qiao email170216384@qq.com) upon reasonable request.
