# Supplementary material for: Glucose uptake in trophoblasts of GDM mice is regulated by the AMPK-CLUT3 signaling pathway
Source: Sci Rep. 2024 May 27;14:12051. doi: 10.1038/s41598-024-61719-7 (PMC11130200; doi:10.1038/s41598-024-61719-7)
Supplement: Supplementary file 1 — Supplementary Figures. [file 41598_2024_61719_MOESM1_ESM.pdf]

peptide blocking experiments

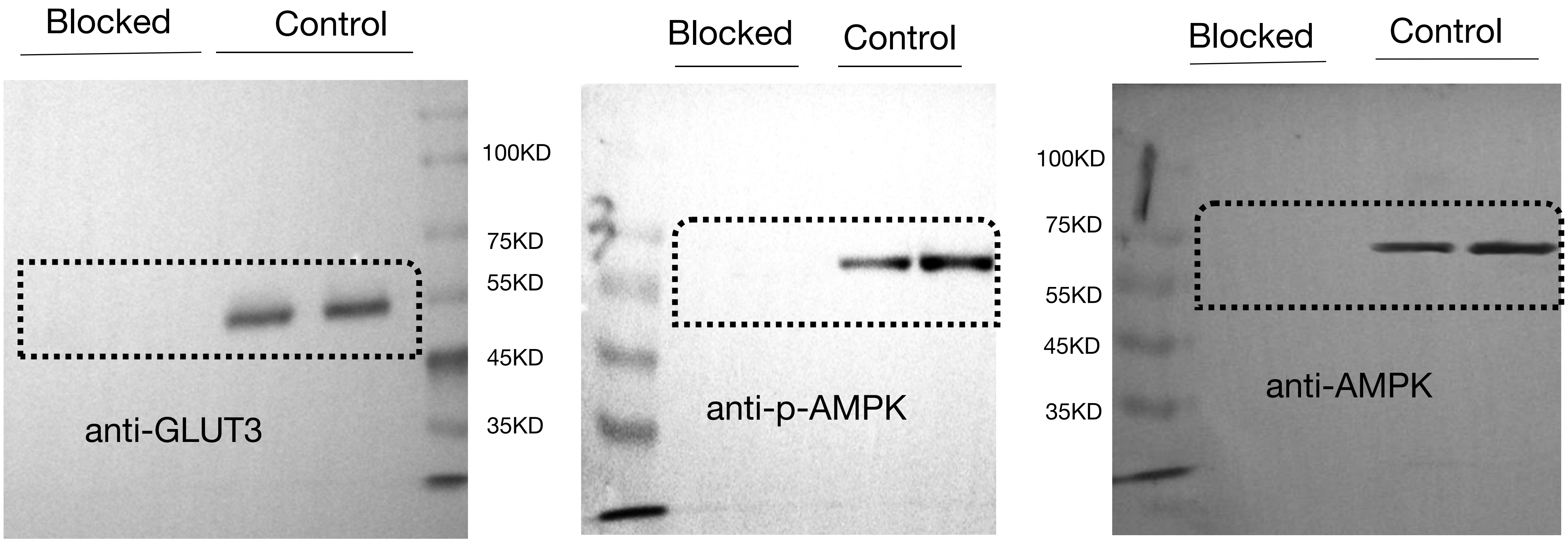

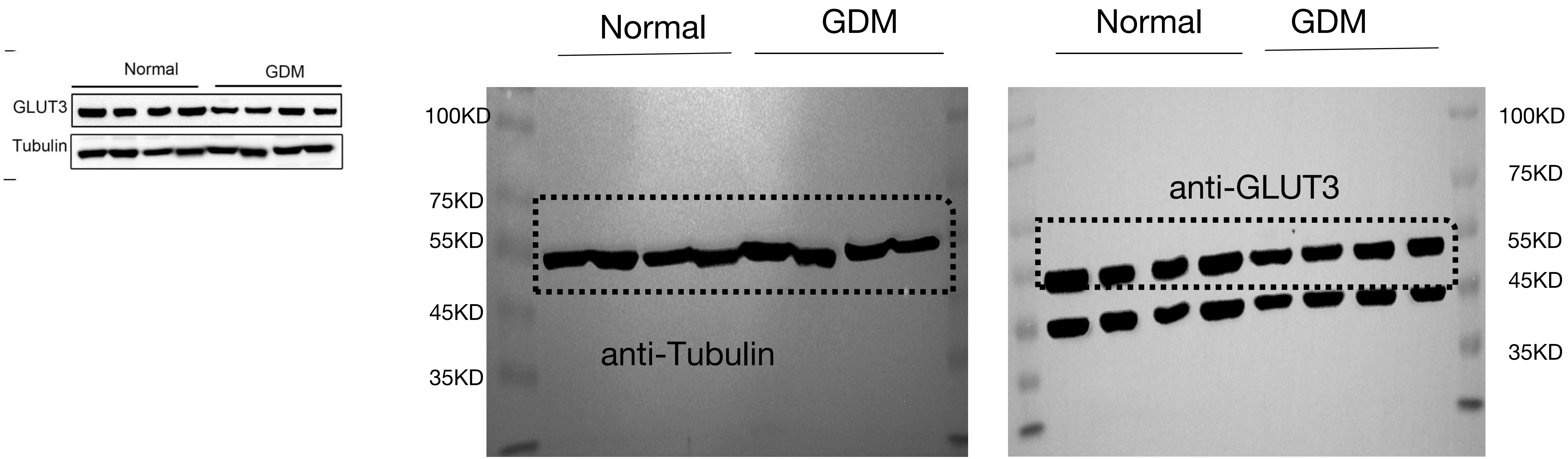

Normal
GDM
GLUT3
Tubulin
Normal
GDM
100KD
75KD
55KD
45KD
35KD
anti-Tubulin
Normal
GDM
anti-GLUT3
100KD
75KD
55KD
45KD
35KD

figure 3 E

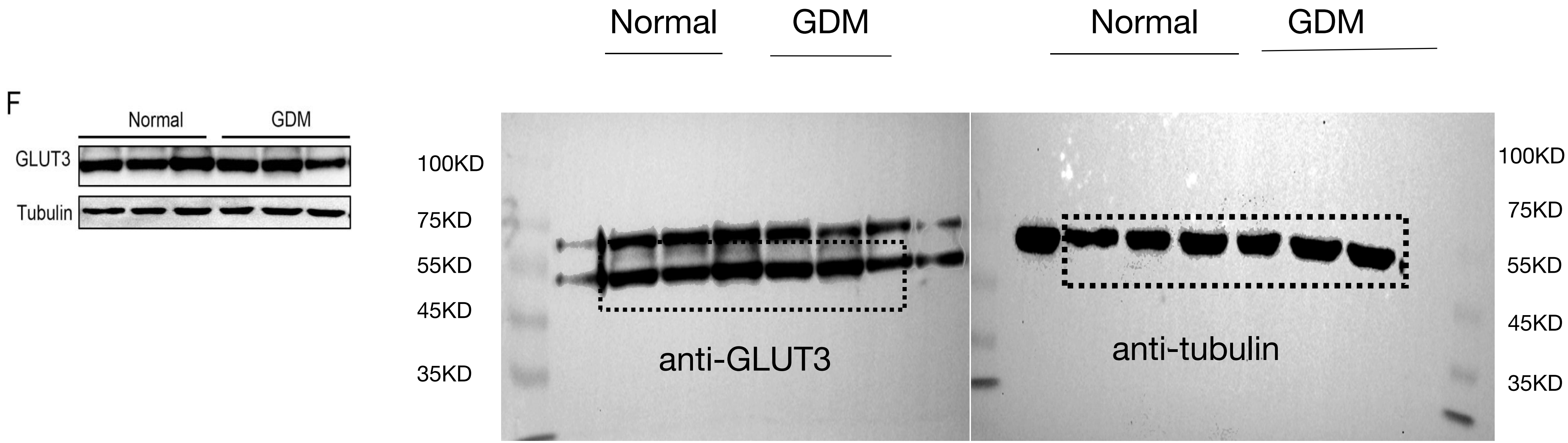
F
Normal
GDM
GLUT3
Tubulin
Normal
GDM
Normal
GDM
100KD
75KD
55KD
45KD
35KD
anti-GLUT3
anti-tubulin
100KD
75KD
55KD
45KD
35KD

figure 3 F

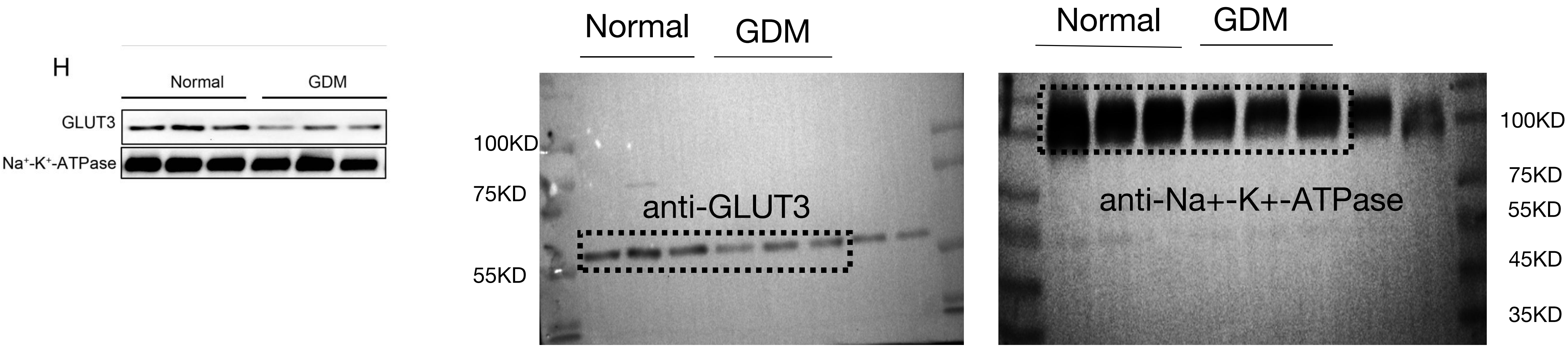
H
Normal
GDM
GLUT3
Na+-K+-ATPase
Normal
GDM
100KD
75KD
55KD
anti-GLUT3
Normal
GDM
100KD
75KD
55KD
45KD
35KD
anti-Na+-K+-ATPase

figure 4 C

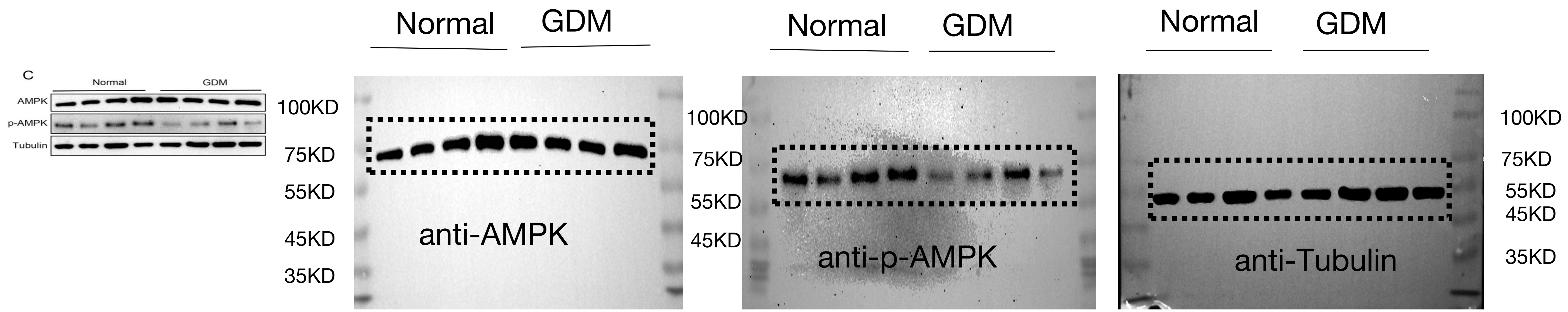
C
Normal
GDM
AMPK
p-AMPK
Tubulin
100KD
75KD
55KD
45KD
35KD
Normal
GDM
anti-AMPK
100KD
75KD
55KD
45KD
Normal
GDM
anti-p-AMPK
Normal
GDM
100KD
75KD
55KD
45KD
35KD
anti-Tubulin

figure 5

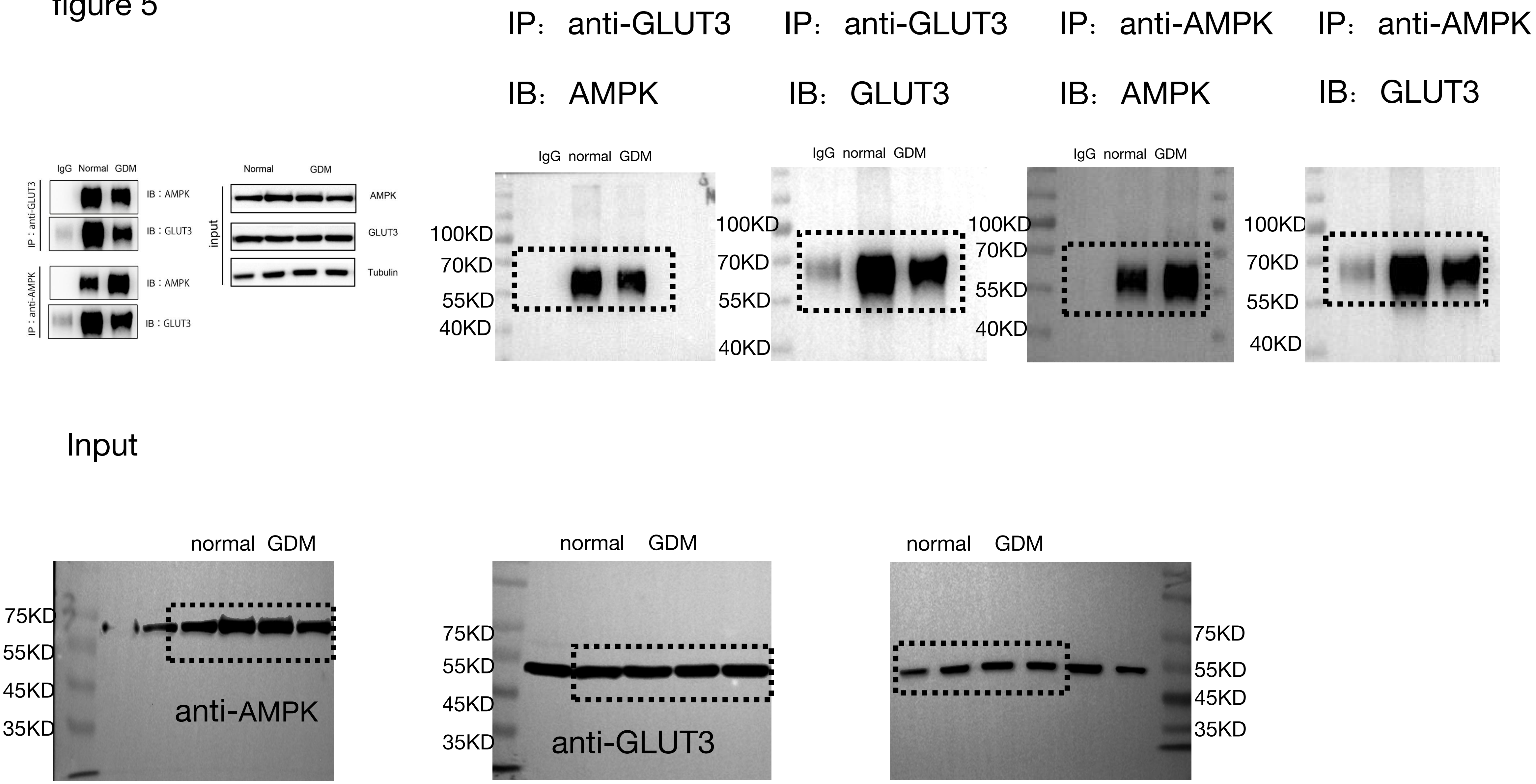
IgG Normal GDM
IP：anti-GLUT3
IB：AMPK
IB：GLUT3
IP：anti-AMPK
IB：AMPK
IB：GLUT3
Normal GDM
input
AMPK
GLUT3
Tubulin
IP：anti-GLUT3
IB：AMPK
IP：anti-GLUT3
IB：GLUT3
IP：anti-AMPK
IB：AMPK
IP：anti-AMPK
IB：GLUT3
IgG normal GDM
100KD
70KD
55KD
40KD
IgG normal GDM
100KD
70KD
55KD
40KD
IgG normal GDM
100KD
70KD
55KD
40KD
100KD
70KD
55KD
40KD
Input
normal GDM
75KD
55KD
45KD
35KD
anti-AMPK
normal GDM
75KD
55KD
45KD
35KD
anti-GLUT3
normal GDM
75KD
55KD
45KD
35KD

figure 7 C

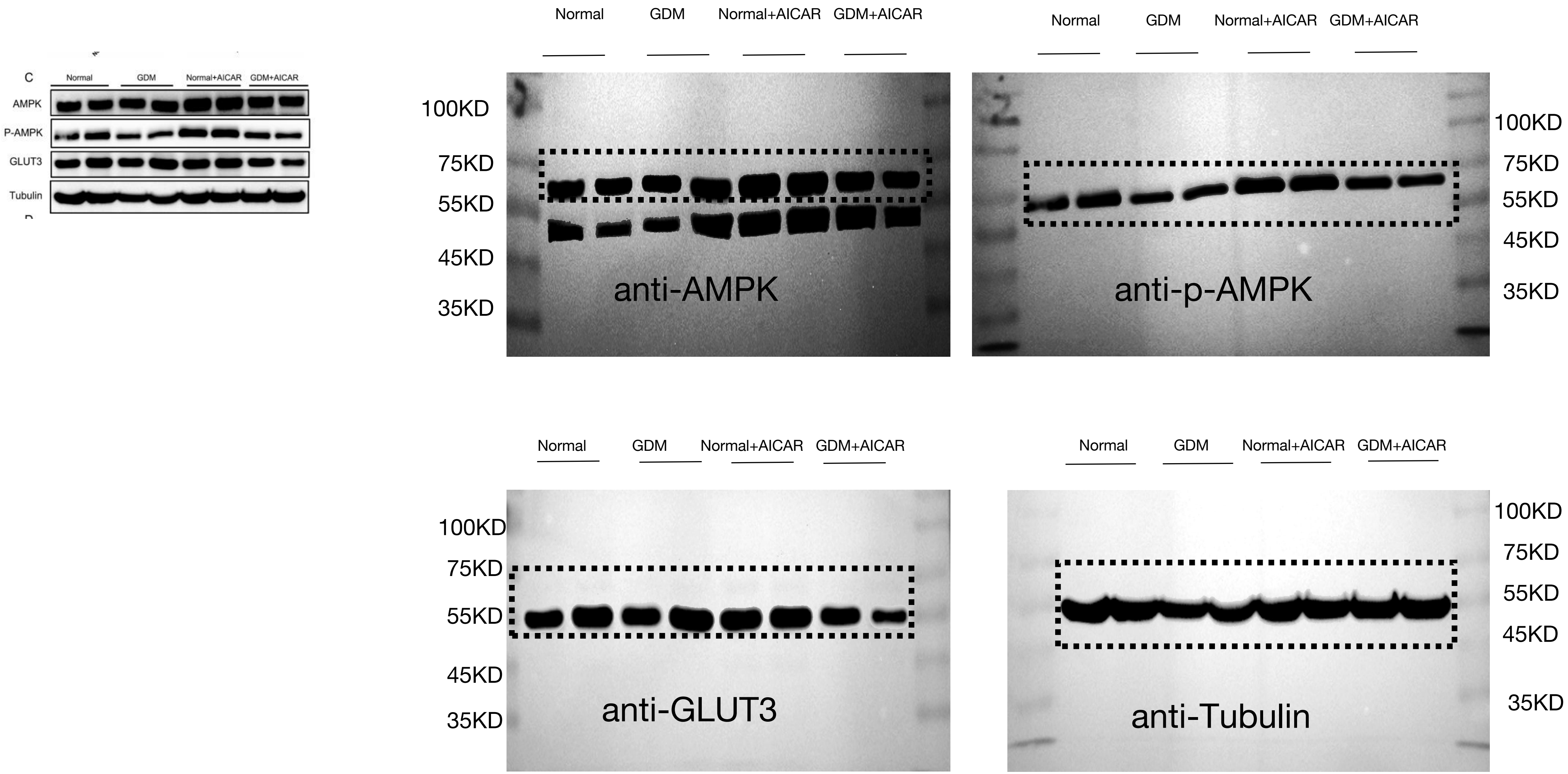
C
Normal
GDM
Normal+AICAR
GDM+AICAR
AMPK
P-AMPK
GLUT3
Tubulin
Normal
GDM
Normal+AICAR
GDM+AICAR
100KD
75KD
55KD
45KD
35KD
anti-AMPK
Normal
GDM
Normal+AICAR
GDM+AICAR
100KD
75KD
55KD
45KD
35KD
anti-p-AMPK
Normal
GDM
Normal+AICAR
GDM+AICAR
100KD
75KD
55KD
45KD
35KD
anti-GLUT3
Normal
GDM
Normal+AICAR
GDM+AICAR
100KD
75KD
55KD
45KD
35KD
anti-Tubulin

figure 7 E

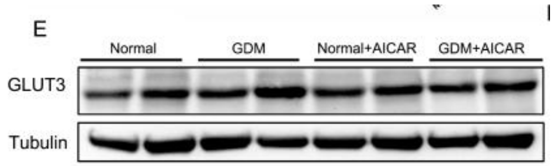
E
Normal
GDM
Normal+AICAR
GDM+AICAR
GLUT3
Tubulin

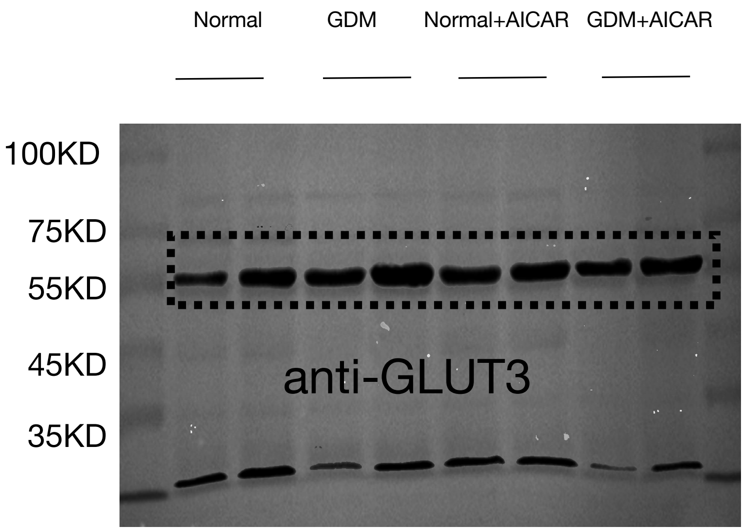
Normal
GDM
Normal+AICAR
GDM+AICAR
100KD
75KD
55KD
45KD
35KD
anti-GLUT3

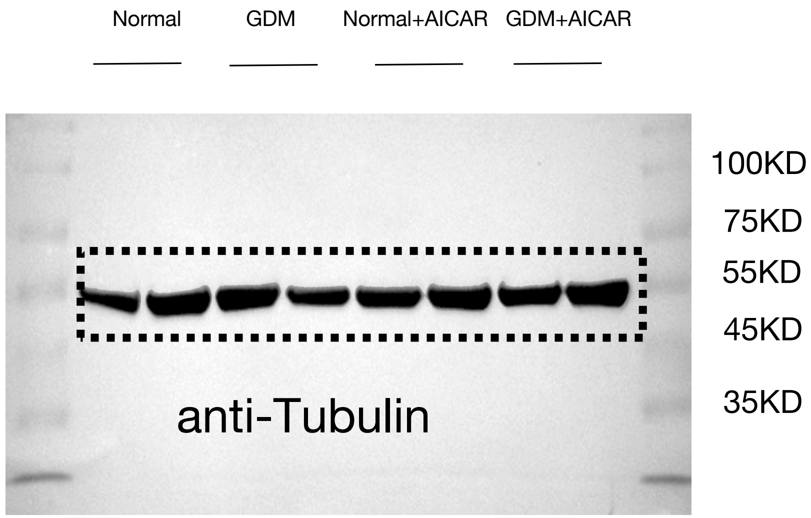
Normal
GDM
Normal+AICAR
GDM+AICAR
100KD
75KD
55KD
45KD
35KD
anti-Tubulin

figure 7 G

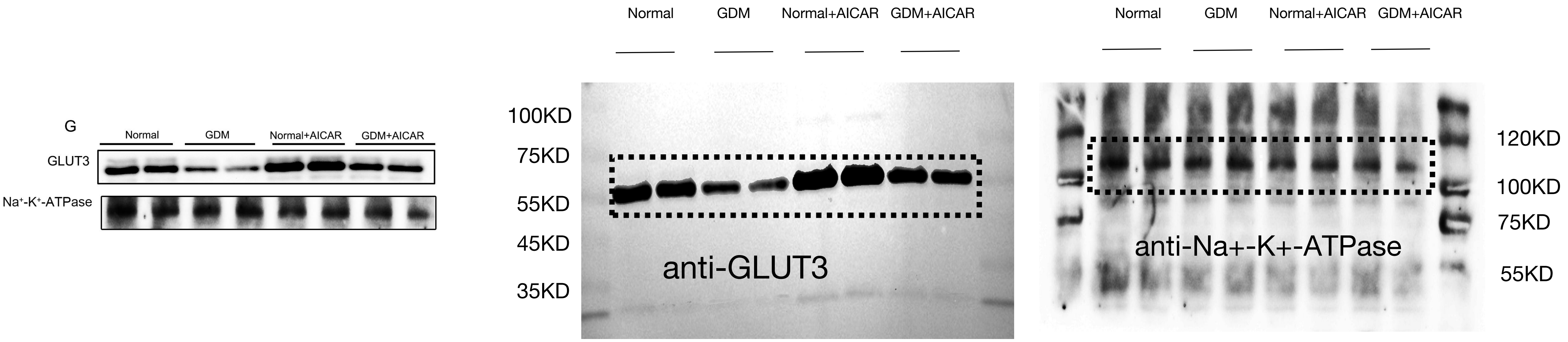
G
Normal
GDM
Normal+AICAR
GDM+AICAR
GLUT3
Na⁺-K⁺-ATPase
Normal
GDM
Normal+AICAR
GDM+AICAR
100KD
75KD
55KD
45KD
35KD
anti-GLUT3
Normal
GDM
Normal+AICAR
GDM+AICAR
120KD
100KD
75KD
55KD
anti-Na+-K+-ATPase
